# Identifying Vital Nodes in Hypergraphs Based on Von Neumann Entropy

**DOI:** 10.3390/e25091263

**Published:** 2023-08-25

**Authors:** Feng Hu, Kuo Tian, Zi-Ke Zhang

**Affiliations:** 1 School of Computer, Qinghai Normal University, Xining 810008, China; qhhuf@163.com; 2 The State Key Laboratory of Tibetan Intelligent Information Processing and Application, Xining 810008, China; 3 College of Media and International Culture, Zhejiang University, Hangzhou 310058, China; 4 Research Center for Digital Communications, Zhejiang University, Hangzhou 310058, China

**Keywords:** hypergraph, high-order line graph, von Neumann entropy, vital nodes, saturation effect

## Abstract

Hypergraphs have become an accurate and natural expression of high-order coupling relationships in complex systems. However, applying high-order information from networks to vital node identification tasks still poses significant challenges. This paper proposes a von Neumann entropy-based hypergraph vital node identification method (HVC) that integrates high-order information as well as its optimized version (semi-SAVC). HVC is based on the high-order line graph structure of hypergraphs and measures changes in network complexity using von Neumann entropy. It integrates s-line graph information to quantify node importance in the hypergraph by mapping hyperedges to nodes. In contrast, semi-SAVC uses a quadratic approximation of von Neumann entropy to measure network complexity and considers only half of the maximum order of the hypergraph’s s-line graph to balance accuracy and efficiency. Compared to the baseline methods of hyperdegree centrality, closeness centrality, vector centrality, and sub-hypergraph centrality, the new methods demonstrated superior identification of vital nodes that promote the maximum influence and maintain network connectivity in empirical hypergraph data, considering the influence and robustness factors. The correlation and monotonicity of the identification results were quantitatively analyzed and comprehensive experimental results demonstrate the superiority of the new methods. At the same time, a key non-trivial phenomenon was discovered: influence does not increase linearly as the s-line graph orders increase. We call this the saturation effect of high-order line graph information in hypergraph node identification. When the order reaches its saturation value, the addition of high-order information often acts as noise and affects propagation.

## 1. Introduction

As an interdisciplinary research field that encompasses big data, machine learning, graph theory, and other related disciplines, network science [1] provides researchers with a novel perspective and approach for studying complex systems in nature and society. It has gained significant popularity and has been widely applied in various domains including social [2], finance [3], biology [4], and transportation [5]. Despite being widely applied to characterize complex systems, ordinary graphs, as a classical research tool in network science, are intrinsically limited in their capacity to describe only the binary interaction relationships between entities. Conversely, in actual complex systems, the existence of collective properties is general, and information activities manifest in multi-body interactions among any number of members. With the deepening development of the network science field and to overcome the limitations of binary interaction systems, hypergraphs have emerged. Meanwhile, researchers have gradually shifted their focus to related theoretical studies of hypergraph structures [6,7], evolution [8,9,10], and dynamics [11,12], while the study of centrality [13,14,15] is also thriving.

A close correlation exists between centrality issues and the recognition of significant nodes, whereby nodes with greater centrality are generally deemed to hold greater importance and tend to have greater influence for information propagation [16] within a network. Among these, vital nodes refer to a special type of node in a network that can have a greater impact on the overall structure and functionality of the network compared to other nodes. The identification of vital nodes, as a key problem in network science research, is crucial for a deeper understanding of network structure and behavior, playing a significant role in various fields. For example, identifying important hubs in transportation networks [17] can help with traffic planning and resource allocation, and further improve the efficiency and safety of transportation networks. In the case of the recent outbreak of the COVID-19 pandemic [18], identifying important patients, close contacts, and carriers in the virus transmission network was of great significance in controlling the spread of the virus and developing scientific prevention and control strategies.

So far, scholars have conducted related research on the task of mining important nodes in hypergraphs, and have proposed some classical methods such as hyperdegree centrality [19] (HDC), closeness centrality [20] (CC), betweenness centrality [21] (BC), and vector centrality [22] (VC), which have provided new perspectives and methods for subsequent research on identifying important nodes in hypergraphs, especially regarding research on entropy. Chen et al. [23] developed the notion of entropy for hypergraphs by using the probability distribution of the generalized singular values of the Laplacian tensor of uniform hypergraphs. They proposed a tensor entropy and proved that it is an extension of the von Neumann entropy for graphs, but it only applies to measuring the uncertainty or disorganization of uniform hypergraphs, and real-world hypergraphs are typically non-uniform. Based on the partial hypergraph structure and using the main sub-matrices associated with the incidence matrix, Bloch et al. [24] generalized the Shannon entropy for hypergraphs and proposed an entropy vector, but this formulation may lose higher-order structural information hidden in the hypergraphs such as nontrivial symmetricity. TUĞAL et al. [25] integrated node degree, hyperedge degree, and hypergraph entropy to quantitatively measure the centrality of nodes and hyperedges, and the method demonstrated applicability in both weighted and unweighted hypergraph structures. Compared to the aforementioned entropies, von Neumann entropy focuses more on the microscopic features of a system and measures the entanglement between nodes and edges from a microscopic perspective. Therefore, this paper conducted research on von Neumann entropy.

Meanwhile, hypergraphs, which accurately and naturally express interactions that go beyond pairwise interactions between entities in complex systems, make the importance of high-order coupling relationships in networks evident. The introduction of high-order information provides new ideas and challenges for research on identifying vital nodes in hypergraphs. To address this, we propose a hypergraph node identification method that integrates higher-order information: high-order von Neumann entropy centrality (HVC). The proposed method captures high-order information through hypergraph high-order line graphs and uses the variation of von Neumann entropy to measure changes in the network’s complexity. The more drastic the change, the greater the impact of the node on the network’s complexity and the more important the node is. At the same time, to balance the complexity and accuracy of the method, we proposed semi-quadratic approximate von Neumann entropy centrality (semi-SAVC) by using quadratic approximations of von Neumann entropy and some high-order information. The performance of the proposed method was comprehensively evaluated using empirical hypergraph datasets from the perspectives of nonlinear propagation influence, robustness, correlation, and monotonicity. We found that the high-order line graph information showed a saturation effect in the task of identifying vital nodes in hypergraphs.

The rest of this paper is organized as follows. Section 2 introduces the basic definitions of hypergraphs, high-order line graphs, and von Neumann entropy. Section 3 describes the baseline methods and hypergraph node identification methods proposed in this paper. In Section 4, the empirical hypergraph datasets used in the experiments are introduced, and the performance of the methods is evaluated comprehensively from the perspectives of influence, correlation, robustness, and monotonicity. Section 5 concludes and provides future research directions.

## 2. Preliminaries

### 2.1. Hypergraph and s-Line Graph

The concept of hypergraphs was first introduced by Berge [26]. A hypergraph, which consists of N nodes and M hyperedges, is defined as an ordered pair $H=\left(V,E\right)$, where $V=\left\{v_{1},v_{2},\ldots ,v_{N}\right\}$ is a finite set of nodes, $v_{i}\left(i=1,2,\ldots ,N\right)$ is a vertex of the hypergraph, $E=\left\{e_{1},e_{2},\ldots ,e_{M}\right\}$, and $e_{j}$ is a hyperedge of the hypergraph, subject to condition:

(1) $$\left\{\begin{array}{c} e_{j}\neq \emptyset \left(j=1,2,\ldots ,M\right)\\ U_{j=1}^{M}e_{j}=V \end{array}\right.$$

For $e_{j}$, its cardinality is denoted as $r_{j}=\left|e_{j}\right|$. The adjacency matrix of H is given by $A_{N\times N}\left(a_{ij}\right)$. If $v_{i}\in e_{k}$ and $v_{j}\in e_{k}$, then $a_{ij}=1$; otherwise, $a_{ij}=0$. Correspondingly, the incidence matrix of hypergraph H is denoted as $B_{M\times N}\left(b_{ji}\right)$. If $v_{i}\in e_{j}$, then $b_{ji}=1$; otherwise, $b_{ji}=0$. Note that the adjacency matrix A is a symmetric matrix and its diagonal elements are all 0.

The sub-hypergraph [27] $H^{\prime }=\left(V^{\prime },E^{\prime }={\left(e_{j}\right)}_{j\in J}\right)$ of hypergraph H, where $V^{\prime }$ is the set of nodes and $E^{\prime }$ is the set of hyperedges, $V^{\prime }\subseteq V$, $e_{j}\in E^{\prime }$, $e_{j}\subseteq V^{\prime }$, $J=\left\{1,2,\ldots ,M\right\}$. Note that a ordinary graph is a special case of hypergraph where the cardinality of any hyperedge is 2.

**Figure 1:**
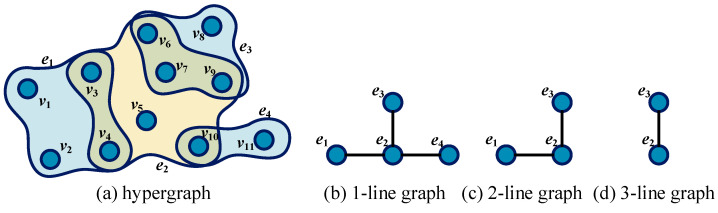
Hypergraph and corresponding s-line graph. (**a**) A hypergraph with 11 nodes and 4 hyperedges; (**b**–**d**) represent the line graph with the order from 1 to 3, respectively.

In a hypergraph, the s-overlap refers to the number of nodes shared between two hyperedges, which is at least s. Figure 1a gives an example of a hypergraph. Let $H=\left(V,E\right)$ be the hypergraph, then $V=\left\{v_{1},v_{2},\ldots ,v_{11}\right\}$ and $E=\left\{e_{1},e_{2},\ldots ,e_{4}\right\}$. According to the definition of the s-overlap, it is obvious that 1-overlap $\left(s=1\right)$ exists between three sets of incident hyperedge pairs (i.e., $\left(e_{1},e_{2}\right)$, $\left(e_{2},e_{3}\right)$, and $\left(e_{2},e_{4}\right))$; the pair of hyperedges $\left(e_{1},e_{2}\right)$ and $\left(e_{2},e_{3}\right)$ also satisfy 2-overlap $\left(s=2\right)$; only one set of hyperedges $\left(e_{2},e_{3}\right)$ has 3-overlap $\left(s=3\right)$.The s-line graph [28] $L_{s}\left(H\right)$ of hypergraph H is a ordinary graph with vertex set $V_{s}=E$. For any $s=1,2,\ldots ,s_{max}$ order line graph, two nodes $e_{i}$ and $e_{j}$ are adjacent if and only if condition $e_{i}\cap e_{j}\geq s$ holds in hypergraph H, where $s_{max}$ is the maximum number of shared nodes among hyperedges. Figure 1b–d shows the s-line graphs $\left(s=1,2,3\right)$ corresponding to the hypergraph in Figure 1a.

### 2.2. Von Neumann Entropy

The concept of von Neumann entropy [29] originally came from quantum mechanics, which is used to describe the uncertainty of a quantum system. With the popularity of quantum mechanics research, it gradually attracted the attention of network science researchers who introduced the concept of von Neumann entropy into the field of network science [30,31] to measure the complexity of networks. Higher entropy values often indicate a higher degree of entanglement between nodes and hyperedges in the network. It seems reasonable to measure the nodes that have a greater impact on the overall structure and behavior of the hypergraph by removing them and measuring the changes in the von Neumann entropy of the hypergraph. From the perspective of quantum mechanics, a system can be described as a quantum state, which is divided into two types: the pure state and mixed state. The pure state is denoted as the state vector $|\psi_{i}〉$, and the weighted statistical set of outer products of pure states is the quantum state. The density operator ρ is a positive semi-definite matrix, defined as follows:

(2) $$\rho =\sum_{i}p_{i}\left|\psi_{i}〉〈\psi_{i}\right|$$

where $p_{i}$ is the probability of the corresponding quantum state. In an ordinary graph $G=\left(x,\varepsilon \right)$ with n nodes and m edges, the density operator ρ is regarded as a measure of the entanglement between the vertex and edge systems [32], which is given by:

(3) $$\rho =\frac{1}{m}\sum_{e_{ij}\in \varepsilon }\left|e_{ij}〉〈e_{ij}\right|=\frac{1}{2m}L\left(G\right)$$

where $|e_{ij}〉=\frac{1}{\sqrt{2}}\left(\left|i〉-\right|j〉\right)$ is a pure quantum state, $|i〉={\left(0,0,\ldots ,1,\ldots ,0\right)}^{T}$ (i.e., |i〉 denotes a column vector where 1 is at the i-thposition, and $L\left(G\right)=D-A$ is the graph Laplacian matrix, where D is the diagonal matrix containing the degrees of the nodes). The von Neumann entropy of a network is defined as the trace and logarithm of the density operator:

(4) $$S\left(\rho \right)=-\mathrm{tr}\left(\rho \ln\rho \right)=-\sum_{i}\lambda_{i}\ln\left(\lambda_{i}\right)$$

where $\lambda_{i}$ denotes the i-th eigenvalue of the density operator ρ. Note that 0ln0=0.

## 3. Method

### 3.1. Baseline Method

HDC: Hyperdegree centrality [19] measures the importance of nodes by the number of incident hyperedges. The more incident hyperedges a node has, the more important it is. The hyperdegree centrality is defined as:

(5) $$HDC\left(v_{i}\right)=\sum_{j=1}^{m}b_{ji}$$

where $b_{ji}$ is the $\left(j,i\right)$-th element of the incidence matrix B of the hypergraph.

CC: The closeness centrality [20] emphasizes the ease or difficulty of a node’s connections with other nodes in the network. It is denoted as the reciprocal of the average distance from a node to all other nodes in the network:

(6) $$CC\left(v_{i}\right)=\frac{N-1}{\sum_{i\neq j}d_{ij}}$$

where N denotes the total number of nodes in the hypergraph, $d_{ij}$ is the shortest distance between node $v_{i}$ and node $v_{j}$, one of common algorithms in solving shortest path problem is Dijkstra algorithm.

VC: The vector centrality [22] of hypergraphs is a vector measure related to the eigenvector centrality in ordinary graphs. First, we project the hypergraph H into a 1-line graph $L_{1}\left(H\right)$ and calculate the eigenvector centrality of each node in $L_{1}\left(H\right)$ (hyperedge in H); let $c\left(e_{j}\right)$ be the eigenvector centrality of any hyperedge $e_{j}\in E$ in H. For any hyperedge with cardinality $r_{j}$ satisfied:

(7) $$2\leq r_{j}\left(j=1,2,\ldots ,M\right)\leq \max\left\{\left|e_{j}\right|;e_{j}\in E\right\}=r_{max}$$

then, the vector centrality of node $v_{i}$ in the hypergraph can be written as:

(8) $$\vec{c_{i}}=\left(c_{i2},c_{i3},\ldots ,c_{ir_{max}}\right)\in {\mathbb{R}}^{r_{max}-1}$$

where:

(9) $$c_{ir_{j}}=\frac{1}{r_{j}}\sum_{\begin{array}{c} e_{j}\in \Gamma_{i}\\ r_{j}=\left|e_{j}\right| \end{array}}c\left(e_{j}\right)$$

$\Gamma_{i}$ denotes the set of incident hyperedges of node $v_{i}$. Finally, the one-norm form expression of node centrality is obtained based on the vector centrality with different hyperedge cardinalities, with larger values indicating greater importance. We thus obtain the vector centrality of node $v_{i}$:

(10) $$VC\left(v_{i}\right)={\left\|\vec{c_{i}}\right\|}_{1}$$

SHC: Sub-hypergraph centrality [33] characterizes the node’s participation in different sub-hypergraphs from a global perspective, denoted as the sum of closed paths of different lengths starting and ending at the node. Similarly, the sub-hypergraph centrality of node $v_{i}$ can also be obtained through algebraic operations on the spectrum of the adjacency matrix:

(11) $$SHC\left(v_{i}\right)=\sum_{j=1}^{N}{\left(\xi_{ij}\right)}^{2}\exp\left(\lambda_{j}\right)$$

where $\lambda_{j}$ denotes the i-th eigenvalue of the adjacency matrix A of the hypergraph, and $\xi_{ij}$ is the i-th element of the eigenvector corresponding to $\lambda_{j}$.

In the subsequent experiments, we chose HDC, CC, VC, and SHC as the baseline methods for comparison. On the one hand, these classical methods have been widely accepted and used in identifying important nodes in hypergraphs, and the comparison of multiple methods can enhance the rigor of the experiment. On the other hand, the four methods approach the problem from different perspectives, which can better highlight the advantages of the proposed method in a comprehensive way.

### 3.2. Identifying Vital Nodes in Hypergraphs

Higher-order information and von Neumann entropy in networks were the focus of the research in this paper. We believe that the incorporation of higher-order information will have a positive effect on identifying important nodes in hypergraphs. Moreover, as suggested in Section 2.2, von Neumann entropy, which originates from quantum mechanics, can effectively capture the degree of entanglement between nodes and hyperedges in a network. Therefore, it is possible that changes in entropy values can be used to better measure the importance of nodes in the network. Therefore, the high-order von Neumann entropy centrality (HVC) was proposed. Considering the increased complexity of the HVC due to the addition of high-order information and complexity issues with von Neumann entropy itself, a method that balances complexity and accuracy has been proposed: semi-quadratic approximate von Neumann entropy centrality (semi-SAVC). The detailed process of the HVC is as follows:

Step 1: For a hypergraph $H=\left(V,E\right)$ containing N nodes and M hyperedges, we first project it into high-order line graphs $L_{s}\left(H\right)$ $\left(s=1,2,\ldots ,s_{max}\right)$, which serve as the basis for high-order information in the centrality method.

Step 2: The change in von Neumann entropy for each s-line graph $\left(1\leq s\leq s_{max}\right)$ after removing a node is calculated using Equations (2) and (3). Let $\Theta \left(L_{s}\left(H\right)\right)$ denote the initial von Neumann entropy of the s-line graph. Since the nodes in the s-line graph correspond to hyperedges in the original hypergraph, the von Neumann entropy of the s-line graph after deleting node $e_{j}$ is denoted as $\Theta \left(L_{s}\left(H\right)/e_{j}\right)$. Therefore, the corresponding change in von Neumann entropy is given by:

(12) $${\Delta \Theta }_{s}\left(e_{j}\right)=\Theta \left(L_{s}\left(H\right)\right)-\Theta \left(L_{s}\left(H\right)/e_{j}\right)$$

The greater the value of ${\Delta \Theta }_{s}\left(e_{j}\right)$, the more significant the impact of removing the node on the complexity of the network, which indicates that the node is more important.

Step 3: Based on the cardinality of hyperedges $r_{j}$, the change in the von Neumann entropy of nodes in the s-line graph is mapped to the nodes in the hypergraph, with smaller weight assigned to nodes with larger cardinality, in other words,

(13) $$\Phi_{s}\left(v_{i}\right)=\sum_{j\in \Gamma \left(v_{i}\right)}\frac{{\Delta \Theta }_{s}\left(e_{j}\right)}{r_{j}}$$

where $\Gamma \left(v_{i}\right)$ is a set of incident hyperedge IDs of node $v_{i}$ in the hypergraph.

Step 4: High-order information of hypergraph is fused, thus finally obtaining the high-order von Neumann entropy centrality:

(14) $$HVC\left(v_{i}\right)=\Phi_{1}\left(v_{i}\right)+\frac{\Phi_{2}\left(v_{i}\right)}{2}+\ldots +\frac{\Phi_{s_{max}}\left(v_{i}\right)}{s_{max}}$$

In the HVC, incorporating high-order information leads to an increase in method complexity. Considering the limitations of the complexity of von Neumann entropy itself, we proposed the semi-quadratic approximate von Neumann entropy centrality (semi-SAVC) approach. The process is similar to the HVC, but differs in that, in Step 2, which only calculates the change in von Neumann entropy of the $s_{max}/2$ order line graph, the compromise of high-order information is a classic technique for improving efficiency [34]. Furthermore, the von Neumann entropy calculation adopts its quadratic approximation [35], which is given by:

(15) $$S\left(\rho \right)=-\mathrm{tr}\left(\rho \ln\rho \right)\approx \frac{\left|x\right|\ln\left(\left|x\right|\right)}{\left|x\right|-1}\mathrm{tr}\left(\rho \left(I_{n}-\rho \right)\right)=\frac{\left|x\right|\ln\left(\left|x\right|\right)}{\left|x\right|-1}\left(1-\frac{1}{4m^{2}}\sum_{v\in x}\left(d{\left(v\right)}^{2}+d\left(v\right)\right)\right)$$

where x denotes the node set of the line graph, $I_{n}$ is the n-order unit matrix, n and m denote the number of nodes and edges, respectively, and $d\left(v\right)$ is the degree of a node, which is the number of edges that the node is adjacent to.

Von Neumann entropy mainly involves solving the problem of matrix eigenvalues and eigenvectors, so calculating the von Neumann entropy requires $O\left(n^{3}\right)$ computational complexity. Specifically, in the HVC method, the mapping from the initial hypergraph to the s-line graph often requires calculating matrices related to s-overlaps, with a time complexity of $O\left(N*M^{2}\right)$ (N and M are the numbers of nodes and hyperedges, respectively). Similarly, calculating the von Neumann entropy also takes up a significant amount of computation time, which occurs after the hypergraph is projected into an s-line graph, and $s_{max}$ is always relatively small compared to the number of hyperedges, so the time complexity of this stage is $O\left(M^{3}\right)$. The overall time complexity is $O\left(\max\left(N*M^{2},\;M^{3}\right)\right)$. The semi-SAVC method uses quadratic approximation of von Neumann entropy, reducing its computational complexity to $O\left(M\right)$, so the overall complexity of the method is $O\left(N*M^{2}\right)$.

## 4. Method Evaluation

### 4.1. Dataset

In this section, we introduce the hypergraph datasets used in the subsequent experiments, which are empirical data from multiple domains. Each dataset has different topological properties, as shown in Table 1.

The Erdos971 dataset was sourced from the famous Pajek dataset [36]. Batagelj et al. [37] analyzed this dataset based on ordinary graphs. However, we constructed a hypergraph based on Erdos’ research collaboration relationships, where nodes denote authors and hyperedges are collaborative publications. The Restaurant and Geometry datasets both came from [38]. In the Restaurant dataset, nodes denote Yelp users and hyperedges are user reviews on different types of restaurants. In the Geometry dataset, nodes denote MathOverflow users, and a group of users who answered the same questions related to geometry are denoted as a hyperedge. The Roget dataset, like Erdos971, also originates from the Pajek dataset. In this dataset, nodes correspond to different categories in Peter Mark Roget’s 1879 edition of the English Thesaurus, while hyperedges are cross-referencing relationships between vocabulary in different categories. The Music-blues dataset was obtained from [39], where Amazon users are denoted as nodes. If different users commented on the same type of music-blues, they would be put into the same hyperedge. The Film-ratings dataset was initially a bipartite graph from the Koblenz Network Collection (KONECT) [40]. We transformed it into a hypergraph based on the relationships between nodes. Nodes denote movies, and if a user rated multiple movies, the movie nodes would be placed in the same hyperedge.

**Figure 2:**
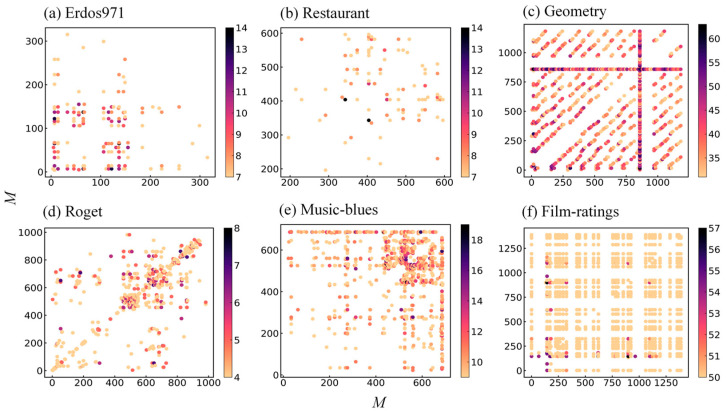
Distribution of the s-overlap between hyperedges in hypergraphs. Both axes represent the hyperedge index, and the color indicates the s-overlap between hyperedges. Maximum s-overlap of the hypergraphs (**a**–**f**) were 14, 14, 63, 8, 19, 57, respectively. To clearly demonstrate the distribution, (**a**–**e**) selected approximately half of the maximum s-overlap, and (**e**) selected the s-overlap starting from s=50.

Next, a detailed analysis of the s-overlap between hyperedges in each dataset was conducted. As shown in Figure 2, the experimental results were consistent with the intuition, where the prevalence of s-overlap between hyperedges decreased gradually as the order s increased (indicated by colors from yellow to black) in the entire hypergraph. Among them, the Roget dataset had the smallest s-overlap with a value of 8, while the Geometry dataset had the largest with 63. It was also found that the distribution of mid-to-high-order s-overlap was more dispersed in Erdos971, Restaurant, Roget, and Music-blues, while high-order s-overlap was heavily concentrated in Geometry and Film-ratings, especially in Film-ratings. This phenomenon may be determined by the practical significance of the hypergraphs. At the same time, it was also noticed that there existed one hyperedge in Geometry that was highly s-overlapped with almost all of the other hyperedges. As a hyperedge denotes a class of geometry problems, this question may be the hottest topic in this field and has attracted many users to participate in answering other questions.

### 4.2. Influence

**Figure 3:**
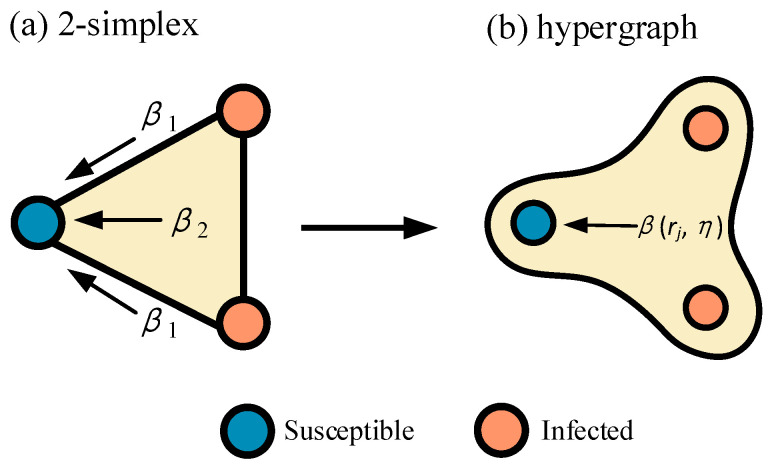
Mapping of the simplicial propagation model to hypergraph the nonlinear propagation model. (**a**) is a simplicial propagation model in 2-simplex with 3 nodes, the infection rate $2\beta_{1}+\beta_{2}$ is related to both the infected nodes and “triangles”. (**b**) represents the hypergraph nonlinear propagation model, and the infection rate $\beta \left(r_{j},\eta \right)$ is variable, where $r_{j}=3$, η=2.

The dynamics of hypergraph propagation provide a solid theoretical foundation to evaluate the identification methods of vital nodes However, existing hypergraph propagation models such as SIS [41], SIR [42], and threshold models [43] often use linear propagation methods. The emergence of nonlinear hypergraph propagation models [44] breaks the linear propagation framework and can better adapt to complex real-life situations and provide more realistic propagation predictions. They considered a more comprehensive range of factors that influence information propagation, capturing differences in propagation between individuals. This process was inspired by the simplex propagation model [45], and the propagation process is illustrated in Figure 3. In a 2-simplex composed of three nodes, a susceptible node is often influenced by other infected nodes and the “triangles”. The infection rate is $2\beta_{1}+\beta_{2}$. If the propagation process is mapped to a hypergraph, it becomes $\beta \left(r_{j},\eta \right)$, where $r_{j}$ is the cardinality of the hyperedge, η denotes the number of infected nodes, and $\eta \leq r_{j}$.

This paper employed a nonlinear propagation evaluation method based on the SIR model of hypergraphs to assess its effectiveness. The nodes in the network have three states, namely susceptible (S), infected (I), and recovered (R); for simplicity, the infection process was modeled nonlinearly while the recovery process was modeled linearly, the specific process is as follows: (I) Select seed nodes based on demand and place them in the I state; (II) At each time step, S state nodes have a probability of $\beta \left(r_{j},\eta \right)=\alpha \eta^{\kappa }$ being infected as I state, where α is an adjustable parameter and κ is a nonlinear exponent (restored to linear when κ=1), and for multiple hyperedge incidents in the same S state node, the infection rate is the simple sum of independent hyperedge infection rates; (III) At each time step, I state nodes have a probability of γ transitioning to the R state; (IV) Repeat steps (II) and (III) until a specified time step t is reached.

**Figure 4:**
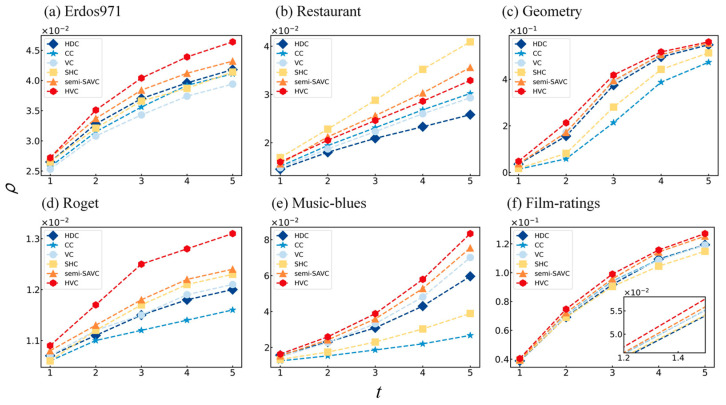
Nonlinear propagation experiment with the top 1% ranked nodes in the hypergraphs. ρ and t represent the influence and time step, respectively. In (**f**), the subplot shows the influence variation of different identification methods on the initial stage of propagation.

Utilizing hypergraph nonlinear propagation, the propagation influence of nodes in different methods serves as compelling evidence for effectiveness. Node influence is measured by the total proportion of I and R state nodes in the network at time step t. As decision-makers often prioritize nodes at the top of the ranking in the network, this experiment compared the hyperdegree centrality (HDC), closeness centrality (CC), vector centrality (VC), sub-hypergraph centrality (SHC), and the methods proposed in this paper, HVC and semi-SAVC, by examining the changes in hypergraph nonlinear propagation influence for the top 1% of ranked nodes among multiple empirical datasets over five time steps. Figure 4 presents the results of 100 repeated simulations with the experimental parameters $\alpha =1\times 10^{-4}$, κ=1.25, γ=0.2.

As indicated in Figure 4, the HVC and semi-SAVC demonstrated superior performance in most datasets (Erdos971, Geometry, Roget, Music-blues, and Film-ratings). Specifically, the proportion of infected and recovered nodes always remained high over the five time steps, suggesting that the top 1% ranked nodes identified by HVC and semi-SAVC were consistently influential at different time steps; this further validates the effectiveness of the proposed methods. Although the effectiveness of the HVC in the Restaurant dataset ranked below the SHC, it still exhibited considerable improvement compared to the HDC, CC, and VC. Additionally, we found that the influence of the HVC was almost always greater than that of the semi-SAVC. This can be attributed to the quadratic approximation of von Neumann entropy, which often results in a loss in accuracy. However, this did not substantially affect the nonlinear propagation influence of the semi-SAVC, which remained higher than the baseline methods. Moreover, compared with the calculation process of HVC, semi-SAVC only considers half of the maximum order $s_{max}$ of the hypergraph corresponding to the line graph, which greatly reduces the computational time, achieving a balance between efficiency and accuracy. Furthermore, we observed that the range of nonlinear propagation influence variations in the Roget dataset was the smallest, which may be closely related to the hypergraph structure. As shown in Table 1, the maximum hyperedge cardinality $\Delta r_{j}$, clustering coefficient C, and efficiency [46] E in the Roget hypergraph were the smallest among the six hypergraph datasets. These three indicators relate to the number of hyperedge nodes, the number of hyper-triangles [47], and the distance between nodes, respectively, indicating that there is insufficient connectivity between nodes in the hypergraph, subsequently affecting the propagation efficiency. Conversely, this can also explain why the variations in influence were more significant in the Geometry and Film-ratings datasets.

**Figure 5:**
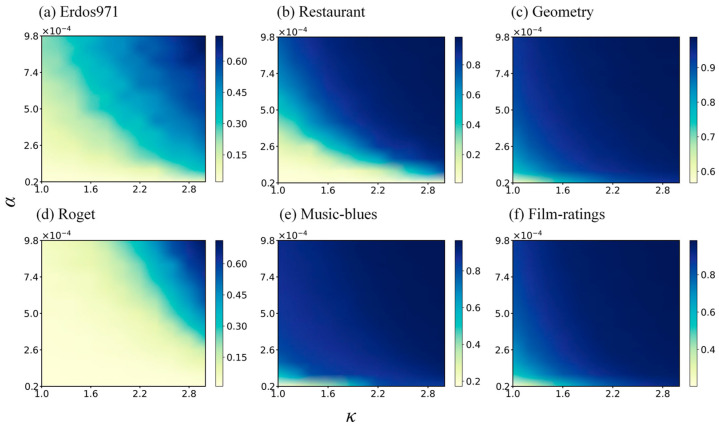
Relationship between the influence of the top 1% ranked nodes identified by the HVC and adjustable parameters α as well as nonlinear exponent κ. The range of variation for α and κ was $2\times 10^{-5}\sim 9.8\times 10^{-4}$ and 1~3, respectively, where the color denotes the node influence value at the fifth time step. The results for other methods were similar.

During the propagation process, the adjustable parameters α and the nonlinear exponent κ play a crucial role. It can be observed from Figure 5 that both the growth of a single parameter and the simultaneous growth of dual exponents have a promoting effect on the node influence. The hypergraph in Geometry, Music-blues, and Film-ratings exhibited rapid influence changes in the early stages of parameter growth, quickly infecting almost all nodes in the network. Conversely, the influence changes in the Erdos971, Restaurant, and Roget hypergraphs were relatively gradual. Similar effects were achieved for different intervals of α and κ. Since the nodes in the Geometry, Music-blues, and Film-ratings hypergraphs were more tightly connected, selecting a smaller value of α may result in a more significant effect.

**Figure 6:**
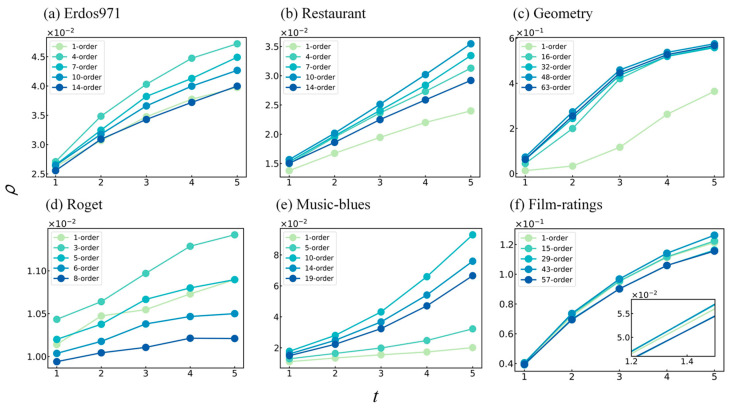
Nonlinear propagation of the top 1% ranked nodes identified by the HVC with different order of the s-line graph in hypergraphs. i-order refers to the maximum order used by the HVC. This is the result of 100 repeated simulations with the experimental parameters $\alpha =1\times 10^{-4}$, κ=1.25, γ=0.2. In (**f**), the subplot shows the influence variation of HVC with different orders of the s-line graph on the initial stage of propagation.

In addition, we observed that in the Restaurant dataset, the influence variations of the HVC and semi-SAVC were similar, but they were distinguishable in the other datasets. This raised our attention. Since the semi-SAVC used a quadratic approximation of von Neumann entropy, it incurred a loss in accuracy. However, its propagation results remained comparable to that of HVC. Could it be that the semi-SAVC adoption of a high-order line graph resulted in an increase in identification accuracy despite halving its order? This led us to the association with the existence of the saturation effect of network information [48,49]. To investigate this, based on the nonlinear propagation model, the influence variations of the top 1% ranked nodes in multiple hypergraph datasets were explored with changes in the order of the high-order line graph corresponding to the hypergraph. Five orders of line graph with similar gradients in the interval $\left[1,s_{max}\right]$ were selected, and each of them was applied to Step 2 of the HVC by replacing $s_{max}$ to identify important nodes in the hypergraphs, and the remaining parameters were set consistently with those in Figure 4. As can be clearly observed from the experimental results in Figure 6, in the six empirical hypergraph datasets, the method with the highest order did not exhibit satisfactory nonlinear propagation results. Methods that fell between the maximum and minimum orders often had greater influence. Although the experiment did not select the line graph order that maximized the influence, it was sufficient to demonstrate our conjecture, namely, the accuracy of identifying vital nodes does not increase with an increase in the line graph order, indicating a saturation effect of higher-order line graph information in identifying vital nodes. Generally, people believe that more high-order information is better, but this saturation effect contradicts intuition. When the line graph order exceeds its saturation point, the addition of other high-order information is likely to act as noise and affect the accuracy of identifying vital nodes. The case in which the HVC is superior to semi-SAVC in Figure 4 may be due to the fact that the quadratic approximation of von Neumann entropy incurs a greater accuracy loss than the addition of high-order information.

### 4.3. Correlation

**Figure 7:**
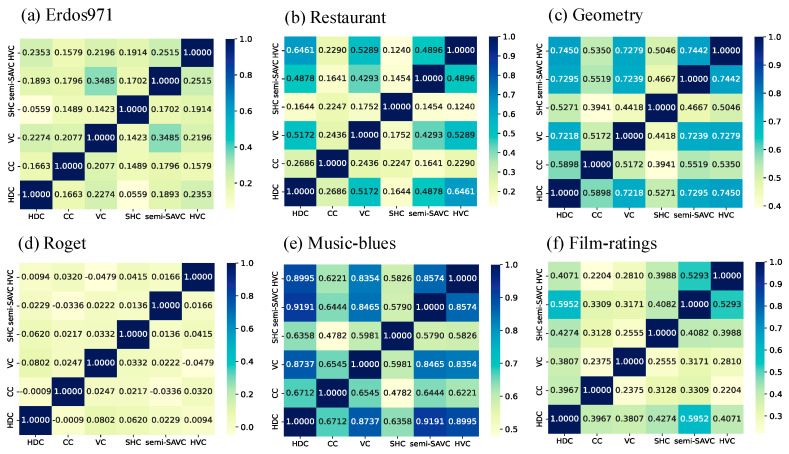
Correlation matrix of vital node identification methods in the hypergraphs. The color of the matrix elements corresponds to the Pearson correlation coefficient values between different methods, ranging from bright green to blue.

In the previous section, the effectiveness of the proposed method was verified through a hypergraph nonlinear propagation model. To further investigate the correlation between identification results from different methods, the Pearson correlation coefficient [50] was introduced. In the natural sciences, the Pearson correlation coefficient is commonly used to measure the correlation between two variables and ranges from −1 to 1. Figure 7 shows the Pearson correlation results between six different vital node identification methods on six empirical hypergraphs.

Firstly, it can be observed that there was consistently high correlation between the HVC and semi-SAVC in most hypergraphs. Additionally, the results in Figure 4 demonstrate the good performance of both methods, which further confirms that the semi-SAVC is considered as a compromise between the accuracy and efficiency of the HVC. At the same time, we found that there is often high correlation between the semi-SAVC, HVC, and HDC, which is determined by the ideas of the proposed methods. The semi-SAVC and HVC are based on the high-order line graph of the hypergraph, and during the mapping process of the von Neumann entropy change caused by isolating hyperedges in the high-order line graph to the importance mapping of the original hypergraph nodes, nodes with a higher hyperdegree tend to have more overlapping mapped values. In addition, the CC and SHC generally have lower correlation with the proposed method. The different focuses of these methods may be the main reason for this phenomenon. The semi-SAVC and HVC focus on measuring the complexity changes of high-order network structures, while the CC and SHC are closely related to the distances between nodes and the information of sub-hypergraphs in the network, respectively.

### 4.4. Robustness

**Figure 8:**
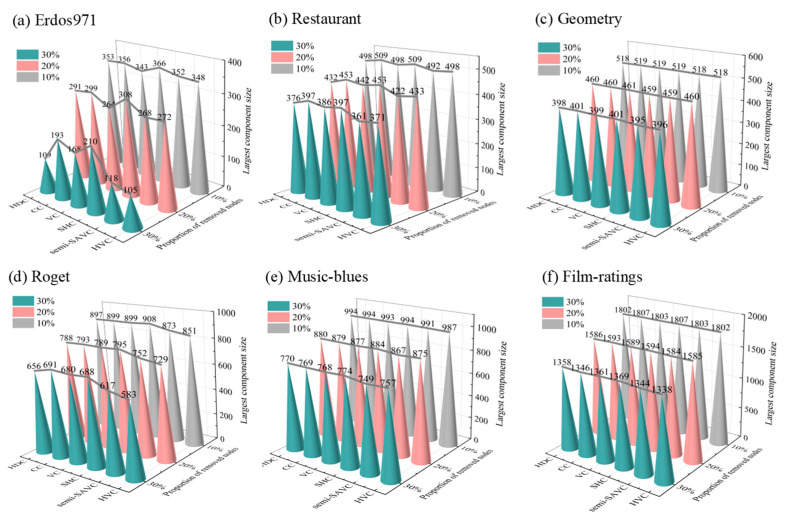
The largest component size after removing the top 10%, 20%, and 30% nodes using different methods in the hypergraphs. The color in the 3-D cone plot represents the proportion of removed nodes. As the removal proportion increases, the largest component size decreases.

Network robustness is a fundamental way to measure the effectiveness of vital node identification methods [51]. It aims to evaluate the ability of a network system to operate normally and maintain good performance in response to various forms of attacks, failures, and abnormal situations. In this section, the effectiveness of the proposed method was evaluated by measuring the change in the size of the maximum connected component of the network after isolating a certain percentage of nodes in the hypergraph. Figure 8 displays the changes in the number of nodes in the largest connected component of the hypergraph after removing the top 10%, 20%, and 30% ranked nodes using six vital node identification methods in six empirical datasets, respectively.

Firstly, it can be observed that in the six hypergraphs, the difference in the largest component size between different methods was not significant when the top 10% nodes were removed. As the removal proportion increased, larger differences tended to occur. This was determined by the multi-body interaction characteristic of the hypergraph, which has a stronger resistance to isolated nodes compared to complex networks. Meanwhile, in the vast majority of hypergraphs (Restaurant, Geometry, Roget, Music-blues, Film-ratings), after removing the top 10%, 20%, and 30% ranked nodes, semi-SAVC and HVC always had the minimum component size, indicating that the top-ranked nodes had a more significant impact on network connectivity and highlighting the effectiveness of the proposed method. In addition, we also found that the changes in the largest component size after removing different proportions of nodes using different methods were extremely similar in the Geometry and Film-ratings hypergraphs. This may be closely related to the network structure. As shown in Table 1, the two hypergraphs had higher values of $\Delta r_{j}$ and $\bar{D}$, indicating that the relationships between nodes were closer and more resistant to destruction, while the opposite was true for the Erdos971 and Roget hypergraphs.

### 4.5. Monotonicity

An effective method for identifying important nodes should not only guarantee the accuracy of the identification results but also emphasize the discriminability of the outcomes. Therefore, we introduced the monotonicity index [52], which is defined as:

(16) $$M\left(R\right)={\left(1-\sum_{r\in R}\frac{N_{r}\left(N_{r}-1\right)}{N\left(N-1\right)}\right)}^{2}$$

where R denotes the node importance ranking table obtained by the node identification method, N is the total number of nodes, and $N_{r}$ is the number of nodes with the same importance level r. $M\left(R\right)\in \left[0,\;1\right]$, the closer the value is to 1, the higher the discriminability of the node importance, and vice versa.

Table 2 compares and analyzes the monotonicity values of the centrality methods based on different entropies in six empirical hypergraphs. HE refers to the node centrality method based on hypergraph entropy mentioned in Section 1 of [25], while PE and ASE are hypergraph important node identification methods based on propagation entropy [53] and adjacency structure entropy [54], respectively. From the data in Table 2, it can be seen that HVC and semi-SAVC had very high importance discriminability in multiple empirical hypergraphs, and the former consistently outperformed the latter, as the latter uses the quadratic approximation of von Neumann entropy and is closely related to node degree. PE and ASE performed moderately, while HE performed the worst. In most networks, there were many nodes with the same degree, which resulted in poor discriminability and lower monotonicity values for these methods.

## 5. Conclusions and Discussion

In this article, we proposed a node identification method (HVC) as well as its optimized version (semi-SAVC). HVC is based on the high-order line graph structure of the hypergraph, which measures the change in network complexity using von Neumann entropy and quantifies node importance in the hypergraph by mapping hyperedges to nodes, incorporating s-line graph information. On the other hand, semi-SAVC uses the quadratic approximation of von Neumann entropy to measure network complexity and considers only half of the maximum order of the s-line graph of the hypergraph. Compared with HVC, it achieves a balance between accuracy and efficiency.

In the six empirical hypergraphs, we compared the performance of the proposed node identification methods from the perspective of propagation influence, correlation, robustness, and monotonicity by evaluating them comprehensively with four baseline methods. Firstly, in the influence evaluation of the methods, we used the latest hypergraph nonlinear propagation model to investigate the relationship between the influence (the proportion of infected and recovered nodes) and time steps. The experimental results showed that the proposed methods always maximized the influence compared to the baseline methods, proving their effectiveness. Meanwhile, we also investigated the influence of adjustable parameters and nonlinear indices in nonlinear propagation on the influence of top-ranked nodes by different methods, and found that both promoted the nonlinear propagation of the hypergraph. In addition, inspired by the above experimental results, we explored the impact of the order of the s-line graph on propagation. The results revealed a crucial non-trivial phenomenon: the node influence does not increase linearly with the order of the s-line graph, which is known as the saturation effect of high-order line graph information in vital node identification in hypergraphs. When the order reaches the saturation value, the addition of high-order information often acts as noise and affects propagation. Then, using the Pearson correlation coefficient, a correlation matrix was constructed to evaluate the correlation of the identification results of different methods. Subsequently, by removing a certain proportion of top-ranked nodes, the proposed methods can minimize the size of the largest component of the hypergraph in most cases, indicating their significant effect of disrupting network structural connectivity. Thus, the methods are effective. Finally, the discriminability of the identification results of the semi-SAVC and HVC was quantitatively evaluated using the monotonicity metric. The data indicate that the proposed methods have high granularity.

Although our work provides some reference value for vital node identification in hypergraphs, this direction still has huge potential. With the development of deep learning technology, the introduction of graph structures and related algorithms in neural network models, graph neural networks [55] have emerged with advantages such as strong representation learning ability and excellent prediction performance. Applying deep learning technologies such as graph neural networks or hypergraph neural networks to identify vital nodes may be a future research direction.

## Figures and Tables

**Table 1:** Topological properties of the hypergraph datasets. $\left|V\right|$ and $\left|E\right|$ represent the number of nodes and hyperedges in a hypergraph, respectively. $\Delta r_{j}$ is the maximum cardinality of hyperedges. $\bar{D}$ represents the average degree of nodes. C represents the clustering coefficient of the corresponding ordinary graph of a hypergraph (represented as a 2-section graph of a hypergraph). E is the efficiency of a hypergraph.

Hypergraph	$\left|V\right|$	$\left|E\right|$	$\Delta r_{j}$	$\bar{D}$	C	E
Erdos971	437	337	36	23.2258	0.7757	0.5268
Restaurant	565	601	43	79.7522	0.5355	0.5503
Geometry	580	1193	230	164.7931	0.8166	0.6367
Roget	1010	997	23	32.2713	0.4587	0.4079
Music-blues	1106	694	83	167.8807	0.6178	0.5551
Film-ratings	2064	1399	151	122.7054	0.7997	0.5043

**Table 2:** Monotonicity values of the identification results of the different entropy methods. The best performance in each hypergraph is highlighted in bold.

Method	Erdos971	Restaurant	Geometry	Roget	Music-Blues	Film-Ratings
HE	0.6520	0.8190	0.8873	0.8164	0.8201	0.6222
PE	0.9625	0.9871	0.9861	0.9918	0.9868	0.8354
ASE	0.9923	0.9979	0.9897	0.9946	0.9909	**0.9102**
semi-SAVC	0.9460	0.9722	0.9579	0.9734	0.8827	0.6578
HVC	**0.9929**	**0.9986**	**0.9947**	**0.9986**	**0.9965**	0.8239

## Data Availability

The data and code used in this work can be accessed via: Github-Tonk/HVC-and-semi-SAVC-for-identifying-vital-nodes: Identifying vital nodes in hypergraphs based on von Neumann entropy.

## References

[1] Barabási A.L. (2013). Network science. Philos. Trans. R. Soc. A Math. Phys. Eng. Sci..

[2] Kong X., Shi Y., Yu S., Liu J., Xia F. (2019). Academic social networks: Modeling, analysis, mining and applications. J. Netw. Comput. Appl..

[3] Veraart L.A.M. (2020). Distress and default contagion in financial networks. Math. Financ..

[4] Yang Y., Qiao S., Sani O.G., Sedillo J.I., Ferrentino B., Pesaran B., Shanechi M.M. (2021). Modelling and prediction of the dynamic responses of large-scale brain networks during direct electrical stimulation. Nat. Biomed. Eng..

[5] Serdar M.Z., Koç M., Al-Ghamdi S.G. (2021). Urban Transportation Networks Resilience: Indicators, Disturbances, and Assessment Methods. Sustain. Cities Soc..

[6] Catalyurek U.V., Boman E.G., Devine K.D., Bozdağ D., Heaphy R.T., Riesen L.A. (2009). A repartitioning hypergraph model for dynamic load balancing. J. Parallel Distrib. Comput..

[7] Zhang Z.K., Liu C. (2010). A hypergraph model of social tagging networks. J. Stat. Mech. Theory Exp..

[8] Guo J.L., Zhu X.Y., Suo Q., Forrest J. (2016). Non-uniform evolving hypergraphs and weighted evolving hypergraphs. Sci. Rep..

[9] Kook Y., Ko J., Shin K. Evolution of real-world hypergraphs: Patterns and models without oracles. Proceedings of the 2020 IEEE International Conference on Data Mining (ICDM).

[10] Hu F., Ma L., Zhan X.-X., Zhou Y., Liu C., Zhao H., Zhang Z.-K. (2021). The aging effect in evolving scientific citation networks. Scientometrics.

[11] Landry N.W., Restrepo J.G. (2020). The effect of heterogeneity on hypergraph contagion models. Chaos: Interdiscip. J. Nonlinear Sci..

[12] Adhikari S., Restrepo J.G., Skardal P.S. (2023). Synchronization of phase oscillators on complex hypergraphs. Chaos: Interdiscip. J. Nonlinear Sci..

[13] Costantini L., Sciarra C., Ridolfi L., Laio F. (2022). Measuring node centrality when local and global measures overlap. Phys. Rev. E.

[14] Zhao S., Sun S. (2023). Identification of node centrality based on Laplacian energy of networks. Phys. A Stat. Mech. Its Appl..

[15] Xie X., Zhan X., Zhang Z., Liu C. (2023). Vital node identification in hypergraphs via gravity model. Chaos Interdiscip. J. Nonlinear Sci..

[16] Xie M., Zhan X.-X., Liu C., Zhang Z.-K. (2023). An efficient adaptive degree-based heuristic algorithm for influence maximization in hypergraphs. Inf. Process. Manag..

[17] Li M., Zhang Q., Deng Y. (2018). Evidential identification of influential nodes in network of networks. Chaos Solitons Fractals.

[18] Chaharborj S.S., Nabi K.N., Feng K.L., Chaharborj S.S., Phang P.S. (2022). Controlling COVID-19 transmission with isolation of influential nodes. Chaos Solitons Fractals.

[19] Battiston F., Cencetti G., Iacopini I., Latora V., Lucas M., Patania A., Young J.-G., Petri G. (2020). Networks beyond pairwise interactions: Structure and dynamics. Phys. Rep..

[20] Aksoy S.G., Joslyn C., Marrero C.O., Praggastis B., Purvine E. (2020). Hypernetwork science via high-order hypergraph walks. EPJ Data Sci..

[21] Yoshida Y. Almost linear-time algorithms for adaptive betweenness centrality using hypergraph sketches. Proceedings of the 20th ACM SIGKDD International Conference on Knowledge Discovery and Data Mining.

[22] Kovalenko K., Romance M., Vasilyeva E., Aleja D., Criado R., Musatov D., Raigorodskii A., Flores J., Samoylenko I., Alfaro-Bittner K. (2022). Vector centrality in hypergraphs. Chaos Solitons Fractals.

[23] Chen C., Rajapakse I. (2020). Tensor entropy for uniform hypergraphs. IEEE Trans. Netw. Sci. Eng..

[24] Bloch I., Bretto A. (2019). A new entropy for hypergraphs. Proceedings of Discrete Geometry for Computer Imagery: 21st IAPR International Conference, DGCI 2019, Marne-la-Vallée, France, 26–28 March 2019.

[25] Tuğal İ., Zeydin P. (2021). Centrality with Entropy in Hypergraphs. Rahva Tek. Ve Sos. Araştırmalar Derg..

[26] Berge C. (1973). Graphs and Hypergraphs.

[27] Berge C. (1984). Hypergraphs: Combinatorics of Finite Sets.

[28] Liu X.T., Firoz J., Lumsdaine A., Joslyn C., Aksoy S., Praggastis B., Gebremedhin A.H. Parallel algorithms for efficient computation of high-order line graphs of hypergraphs. Proceedings of the 2021 IEEE 28th International Conference on High Performance Computing, Data, and Ana-lytics (HiPC).

[29] Nielsen M.A., Chuang I. (2010). Quantum Computation and Quantum Information.

[30] Minello G., Rossi L., Torsello A. (2018). On the von Neumann entropy of graphs. J. Complex Networks.

[31] Sarkar R., Dutta S., Banerjee S., Panigrahi P.K. (2021). Phase squeezing of quantum hypergraph states. J. Phys. B At. Mol. Opt. Phys..

[32] Severini S., De Beaudrap N., Giovannetti V., Wilson R. (2016). Interpreting the von Neumann entropy of graph Laplacians, and coentropic graphs. A Panor. Math. Pure Appl..

[33] Estrada E., Rodríguez-Velázquez J.A. (2006). Subgraph centrality and clustering in complex hyper-networks. Phys. A Stat. Mech. its Appl..

[34] Zhang H., Zhong S., Deng Y., Cheong K.H. (2021). LFIC: Identifying influential nodes in complex networks by local fuzzy in-formation centrality. IEEE Trans. Fuzzy Syst..

[35] Wang J., Wilson R.C., Hancock E.R. (2021). Network edge entropy decomposition with spin statistics. Pattern Recognit..

[36] Batagelj V., Mrvar A. (2006). Pajek Datasets. https://vlado.fmf.uni-lj.si/pub/networks/data/.

[37] Batagelj V., Mrvar A. (2000). Some analyses of Erdos collaboration graph. Soc. Networks.

[38] Amburg I., Veldt N., Benson A.R. (2022). Diverse and experienced group discovery via hypergraph clustering. Proceedings of the 2022 SIAM International Conference on Data Mining (SDM).

[39] Ni J., Li J., McAuley J. Justifying recommendations using distantly-labeled reviews and fine-grained aspects. Proceedings of the 2019 Conference on Empirical Methods in Natural Language Processing and the 9th International Joint Conference on Natural Language Processing (EMNLP-IJCNLP).

[40] Kunegis J. Konect: The koblenz network collection. Proceedings of the 22nd International Conference on World Wide Web.

[41] Suo Q., Guo J.L., Shen A.Z. (2018). Information spreading dynamics in hypernetworks. Phys. A Stat. Mech. Its Appl..

[42] Zhang Z., Mei X., Jiang H., Luo X., Xia Y. (2023). Dynamical analysis of Hyper-SIR rumor spreading model. Appl. Math. Comput..

[43] de Arruda G.F., Petri G., Moreno Y. (2020). Social contagion models on hypergraphs. Phys. Rev. Res..

[44] St-Onge G., Iacopini I., Latora V., Barrat A., Petri G., Allard A., Hébert-Dufresne L. (2022). Influential groups for seeding and sustaining nonlinear contagion in heterogeneous hypergraphs. Commun. Phys..

[45] Jhun B. (2021). Effective epidemic containment strategy in hypergraphs. Phys. Rev. Res..

[46] Criado R., Romance M., Vela-Pérez M. (2010). Hyperstructures, a new approach to complex systems. Int. J. Bifurc. Chaos.

[47] Behague N.C., Bonato A., Huggan M.A., Malik R., Marbach T.G. (2023). The iterated local transitivity model for hypergraphs. Discret. Appl. Math..

[48] Li H.J., Wang L., Bu Z., Cao J., Shi Y. (2021). Measuring the network vulnerability based on markov criticality. ACM Trans. Knowl. Discov. Data (TKDD).

[49] Wang X., Slamu W., Guo W., Cao J., Shi Y. (2022). A novel semi local measure of identifying influential nodes in complex networks. Chaos Solitons Fractals.

[50] Cohen I., Huang Y., Chen J., Benesty J. (2009). Pearson Correlation Coefficient. Noise Reduction in Speech Processing.

[51] Xu X., Zhu C., Wang Q., Zhu X., Zhou Y. (2020). Identifying vital nodes in complex networks by adjacency information entropy. Sci. Rep..

[52] Wang M., Li W., Guo Y., Peng X., Li Y. (2020). Identifying influential spreaders in complex networks based on improved k-shell method. Phys. A Stat. Mech. its Appl..

[53] Wu Y.H., Tian K., Li M.D., Hu F. Important node recognition in hypernetworks nased on node propagation entropy. J. Comput. Eng. Appl. 2023, 60, 1–11..

[54] Zhou L.N., Chang X., Hu F. (2022). Using adjacent structure entropy to determine vital nodes of hypernetwork. J. Comput. Eng. Appl..

[55] Zhou J., Cui G., Hu S., Zhang Z., Yang C., Liu Z., Wang L., Li C., Sun M. (2020). Graph neural networks: A review of methods and applications. AI Open.

